# Evaluation of implementation outcomes of an integrated group postpartum and well-child care model at clinics in Malawi

**DOI:** 10.21203/rs.3.rs-2515043/v1

**Published:** 2023-02-07

**Authors:** Ashley Gresh, Janet Mambulasa, Nellie Ngutwa, Ellen Chirwa, Esnath Kapito, Nancy Perrin, Nicole Warren, Nancy Glass, Crystal L. Patil

**Affiliations:** Johns Hopkins School of Nursing; Kamuzu University of Health Sciences; Kamuzu University of Health Sciences; Kamuzu University of Health Sciences; Kamuzu University of Health Sciences; Johns Hopkins School of Nursing; Johns Hopkins School of Nursing; Johns Hopkins School of Nursing; University of Illinois at Chicago

**Keywords:** postpartum, maternal and child health services, shared medical appointments

## Abstract

**Background:**

Persistently elevated rates of maternal and infant mortality and morbidities in Malawi indicate the need for increased quality of maternal and well-child care services. The first-year postpartum sets the stage for long-term health for the childbearing parent and infant. Integrated group postpartum and well-child care may improve maternal and infant health outcomes. The purpose of this study was to examine implementation outcomes for this model of care.

**Methods:**

We used mixed methods to examine implementation outcomes of integrated group postpartum and well-child care. We piloted sessions at three clinics in Blantyre District, Malawi. During each session we evaluated fidelity using a structured observation checklist. At the end of each session, we administered three surveys to health care workers and women participants, the Acceptability of Intervention Measure, the Intervention Appropriateness Measure, and the Feasibility of Intervention Measure. Focus groups were conducted to gain greater understanding of people’s experience with and evaluation of the model.

**Results:**

Forty-one women with their infants participated in group sessions. Nineteen health care workers across the three clinics co-facilitated group sessions, 9 midwives and 10 health surveillance assistants. Each of the 6 sessions was tested once at each clinic for a total of 18 pilot sessions. Both women and health care workers reported group postpartum and well-child care was highly acceptable, appropriate, and feasible across clinics. Fidelity to the group care model was high. During each session as part of structured observation the research team noted common health issues, the most common one among women was high blood pressure and among infants was flu-like symptoms. The most common services received within the group space was family planning and infant vaccinations. Women reported gaining knowledge from health promotion group discussions and activities. There were some challenges implementing group sessions.

**Conclusion:**

We found that clinics in Blantyre District, Malawi were able to implement group postpartum and well-child care with fidelity and that it was highly acceptable, appropriate, and feasible to women and health care workers. Due to these promising results, we recommend future research examine the effectiveness of the model on maternal and child health outcomes.

## Background

Maternal mortality rates in low- and middle-income countries (LMICs) remain alarmingly high (1). For every woman who dies from pregnancy-related causes many more experience life-threatening complications (2). Maternal morbidity and mortality lead to poor short- and long-term maternal and infant health outcomes (2). Globally, Malawi has some of the highest rates of maternal and infant mortality at 439 maternal deaths per 100,000 live births and 42 infant deaths per 1,000 live births (3). While rates are unknown in Malawi, it is estimated that one in three women experience a maternal morbidity (4). These are mostly preventable outcomes that may be improved through improvements in quality of care (5–7).

The postpartum period, defined here from the time of birth through the first year, is a critical time to reduce maternal and infant morbidity and mortality and is often neglected in the care continuum (8). The World Health Organization (WHO) recommends that every mother and baby should have at least four postpartum visits within the first six weeks of giving birth (9). Currently there are low rates of postpartum care attendance within the first six weeks postpartum (3), showing that women’s needs are unmet during this critical period in their life course. Moreover, it is well established that the first year after childbirth is a period of not only physical recovery but is an important time to identify and manage health and social challenges including psychosocial adaptations and transitions to a parental role, that make women and children vulnerable for poor health outcomes (10). However, there is no standardized package of care for the first year postpartum.

Group care that integrates both postpartum and well-child care offers a promising strategy to reduce both maternal and infant morbidities and mortalities (11). A large body of rigorous evidence shows effectiveness and feasibility of bringing group antenatal care (ANC) to scale (12, 13). Group ANC is acceptable, feasible, and improved outcomes in several countries in the African continent including health literacy, antenatal and postpartum attendance, the number of health facility births, and breastfeeding practices (14–16). Extending group care into the postpartum period holds promise for standardizing a package of care for quality postpartum and well-child care services.

CenteringParenting is one such group care model with three core components including healthcare in a group space, interactive learning, and community building that provides a structure to build capacity and enable clinicians to provide efficient and effective care (17). In CenteringParenting, 6–8 women with similarly aged infants receive care together for up to 2 years. Each visit is 2 hours with the first 30–45 minutes devoted to standard clinical health assessments for the infant and childbearing parent and selfcare (measuring their own infant’s weight and length and taking the parent’s blood pressure and weight). After health assessments, co-facilitators (often a physician or midwife and nurse or community health worker) facilitate 75–90 minutes of interactive health promotion activities. Despite the growing evidence-base and associated positive results for CenteringParenting and other similar group care models (18), it has not been widely implemented in LMICs. In addition, most of these group care models prioritize the infant. However, given the high rates of maternal and infant mortality and morbidity and sub-optimal postpartum care attendance rates in LMICs, there is an urgent need to provide care for both the childbearing parent and infant.

To fill this gap, we adapted the CenteringParenting model to be an integrated postpartum and well-child group care model that balances the focus on the childbearing parent and the infant. As detailed in a forthcoming paper (19), our Centering-based and integrated group postpartum and well-child care model prototype was co-created with women and health care workers using a five-step human-centered design (HCD) approach to produce a model that is adapted to the Malawian context (20). Briefly, we created a 6-visit model to be implemented over the first 12 months postpartum that included Malawi-specific health promotion content for the dyad. For example, this included HIV-prevention messages for the women and alignment with the Malawian child vaccination schedule. The adapted Centering-based model includes 6 sessions, an implementation schedule, a facilitator’s guide providing a curriculum for each group session with prioritized health promotion content and learning activities, and clinical assessment guidelines.

The last step in the human-centered design process is to evaluate the implementation of this Centering-based integrated group postpartum and well-child care prototype. Evaluating implementation success is important step to introducing a new model of care within a health system and a necessary precondition for realizing the desired impact of group postpartum and well-child care on maternal and child health outcomes (21). Therefore, the purpose of this study was to examine implementation outcomes (acceptability, appropriateness, feasibility, and fidelity) of the adapted Centering-based integrated group postpartum and well-child care model at clinics in Blantyre District, Malawi.

## Methods

### Study design and setting

We used a mixed methods design to examine implementation outcomes of the integrated 6-visit group postpartum and well-child care model at clinics in Blantyre District, Malawi. Using quantitative surveys, structured observation checklists and qualitative methods (focus groups and field notes) we evaluated if women and health care worker facilitators felt that this model of care was acceptable, appropriate, and feasible and whether health care workers could implement it with fidelity to the model.

We selected three government-run clinics representing a range of variation in size and staffing to pilot and evaluate the groups. All three clinics are taking part in the ongoing study, “Group Antenatal Care: Effectiveness for Maternal/Infant and HIV Prevention Outcomes and Contextual Factors Linked to Implementation Success in Malawi,” (ANC Trial, UIC IRB #00403255), and have been implementing and sustaining group ANC since 2019 (22).

### Sample And Recruitment

We recruited postpartum women and infant dyads using convenience sampling at each clinic. We recruited women at clinics attending either postpartum or well-child visits with an infant less than 12 months old. Women were ineligible if 1) they had a marked cognitive impairment that would prevent providing informed consent, 2) they did not speak and understand Chichewa, or 3) were unable or unwilling to attend two group postpartum and well-child care sessions over the course of one to two weeks.

We used purposive sampling to recruit health care worker facilitators. These included midwives and health surveillance assistants (HSAs) who were hired and trained to facilitate the integrated group care model. If interested, midwives and HSAs were screened eligible if they worked at one of the three clinics and had at least one year of experience working in postpartum and/or well-child care. Midwives and HSAs were ineligible if they 1) they did not read/speak Chichewa and/or English at a grade 8 level, or 2) they were unable or unwilling to facilitate multiple sessions over a four-week period. Since data were also collected from the group facilitators, research assistants obtained written consent.

### Procedures

#### Training for facilitators.

Prior to implementation of the integrated group care model, all facilitators participated in experiential training on the 6-visit integrated group care sessions (23). They were given a copy of the facilitator’s guide detailing the structure of each session, clinical assessment guidelines, and suggested interactive learning activities to deliver health promotion content.

#### Integrated group care model pilot session implementation.

Each of the 6 sessions was tested three times, once at each clinic, for a total of 18 pilot sessions. All integrated group care sessions occurred on site at the clinic in space designated by the midwife in charge at each facility. Each session was designed to be 2 hours in length with women/infant dyads and 2 co-facilitators, a midwife and HSA. Each session began with health assessments of both the woman and infant, followed by interactive health promotion activities, and ending with provisioning services e.g., infant vaccinations. Research assistants observed each session to assess model fidelity. Women/infant dyads were asked to attend a total of 2 sessions on days scheduled by the co-facilitators. Both women and health care workers facilitators received compensation for their participation. De-briefings with the research team occurred after each session, allowing for suggested refinements of the session’s content and delivery.

#### Data collection.

Before each session began, demographic data were collected for the women/infant dyads and facilitators. We then used mixed methods including participant and facilitator surveys, a fidelity observation checklist, detailed field notes, and focus groups with participants to assess four implementation outcomes: acceptability, appropriateness, feasibility, and fidelity. Research assistants administered surveys at the end of each session within the group space in the local language, Chichewa. Each survey took approximately 10 minutes to complete. Separate focus groups were also conducted at the end of each session in Chichewa by research assistants for women and then for facilitators.

#### Implementation Outcomes Survey: Acceptability, Appropriateness, and Feasibility.

The Acceptability of Intervention Measure (AIM), Intervention Appropriateness Measure (IAM), and Feasibility of Intervention Measure (FIM) developed by Weiner et al.(24) were administered at the end of each session to both women and facilitators. Each of these measures were designed to be as general as possible to allow for adapting each measure, in this case for the group postpartum and well-child care model (24). For example, an item of the AIM was *“Group postpartum/well-child care meets my approval”* The AIM evaluates whether group postpartum and well-child care is perceived as agreeable or satisfactory based on their direct experience of each session (21). The IAM evaluates the perceived relevance or fit of group postpartum and well-child care to address health care needs (21). And the FIM evaluates the extent to which group postpartum and well-child care can be successfully carried out in the Malawian context (21). Each measure contains 4 items with a five-point Likert scale, where 1 = Strongly disagree, 2 = Disagree, 3 = Neither Agree nor Disagree, 4 = Agree, and 5 = Strongly Agree. Therefore, the higher the mean value, the more acceptable, appropriate, or feasible the group care model was felt to be. All three of these measures are psychometrically validated in multiple languages to assess the implementation outcome measures: acceptability, appropriateness, and feasibility (24, 25). The reported Cronbach alphas for the scales were 0.85 for acceptability, 0.91 for appropriateness, and 0.89 for feasibility (24). The measures were translated from English to Chichewa by a committee consisting of researchers, a bilingual midwife, and two lay people (26).

#### Fidelity to the Group Care Model.

A fidelity observation checklist was adapted from the existing group ANC model and administered by trained research assistants to assess fidelity to the core components and implementation of the postpartum and well-child care model (22). This checklist was adapted for use in this study and was scored across eight domains (Table 1). While to date, no standardized benchmarks exist for measuring fidelity, in a systematic review of fidelity measures with a focus on self-management and health promotion interventions, the review examined the influence of implementation on program outcomes and found positive effects at 60% model fidelity, and few studies achieved greater than 80% model fidelity (27). So, we set the threshold for achieving fidelity to 80–100% for each domain and 80% for the total fidelity score.

In addition to the scored items, research assistants used the checklist to create detailed field notes and observations on the flow of the sessions, from health assessments to group activities, participant responses and engagement with activities. Observations also assessed co-facilitators’ engagement and facilitation skills in delivering health promotion content in each session. Additionally, research assistants recorded attendance and availability of space and equipment in the detailed field notes. If a session was observed by more than one team member, after each session they reviewed their responses with the team and reconciled any differences through group discussion to build consensus.

#### Focus groups.

Each focus group lasted approximately 30 minutes and included open-ended questions to further assess acceptability, appropriateness, feasibility, explore implementation facilitators and barriers, and capture any recommendations and suggested revisions to the content and structure of the model. Open ended questions also sought to explore if there was any knowledge gained by women by attending group sessions.

### Data analysis

#### Quantitative methods

The AIM, IAM, FIM, and fidelity observation checklist were analyzed using descriptive statistics using Stata 17. The AIM, IAM, FIM were analyzed by calculating the aggregated mean scores at each of the three clinics. A t-test was used to assess if there were differences between those facilitating versus those receiving group. The fidelity observation checklist was also analyzed in total and at the clinic level. For each of the 6 sessions, the percent of items meeting the threshold was determined and the mean percent fidelity across the 6 sessions computed for using the first 5 items of the fidelity measure to get a score for each clinic. The mean of the last 3 item scores was taken for each session and then averaged across session for each clinic.

#### Qualitative methods

After all 18 group sessions were completed, and before data analysis began, the research team met to resolve any conflicts in the data. Then the detailed field notes from the fidelity observation checklist and focus groups were collated and analyzed separately using content analysis methods (28). Field notes were written in English and coded both deductively and inductively by two research team members. Focus group notes were taken in English with research assistants conducting simultaneous translation while documenting in real time and coded both deductively and inductively by two research team members. A codebook for each set of notes was developed with deductive codes based on the defined implementation outcomes: acceptability, appropriateness, feasibility, and fidelity. New codes were created as new themes emerged. We followed the consolidated criteria for reporting qualitative research (COREQ) (29).

### Ethics

This study was approved by the Institutional Review Boards at Johns Hopkins University School of Nursing (IRB #00245018) and Kamuzu University of Health Sciences (IRB #P06/21/3341).

## Results

### Participant and group characteristics

Forty-one women with their infants consented to participate in this study and women ranged in age from 16 to 40 years old with infants ranging from 1 day to 10 months old. Characteristics of women and infant participants are summarized in Table 2. A total of nineteen facilitators across the three clinics co-facilitated group sessions, 9 midwives and 10 HSAs. Facilitators were health care workers with experience working in postpartum and/or well-child care from 1 to 23 years.

Group sizes ranged from 2 to 6 women/infant dyads per session and each session was conducted by two co-facilitators either a midwife and an HSA or two midwives. Of the 18 sessions, 1 included 6 dyads, 12 included 5 dyads, 3 included 4 dyads, 1 included 3 dyads, and 1 included 2 dyads. Each group lasted between 90 to 120 minutes in length.

### Acceptability, Appropriateness, and Feasibility

Both women and facilitators reported that integrated group postpartum and well-child care was highly acceptable, appropriate, and feasible across clinics (Table 3). Combined mean scores for all measures of both women and facilitators ranged from 4.56 (SD .90) to 4.76 (SD .56) indicating they both agreed or strongly agreed that group postpartum and well-child care was acceptable, appropriate, and feasible. Facilitators consistently had higher scores across all three measures. There was a significant difference when comparing the mean scores between women and facilitators for the IAM (p = .028) and FIM (p = .002), but not for the AIM (p = .361)

In focus groups, when asked about the acceptability of group sessions, many women stated that they enjoyed their experience in the group and hoped that groups would continue being offered in the postpartum period. Some women asked when they could return to attend groups at the clinic and expressed a desire to be advocates in their community to promote group care. A woman from Clinic C said, “*What we have learned we can use it at home to be community advocates.”* Similarly, a woman from Clinic A expressed her appreciation for the model when she said, “I *am so thankful to be a part of the group and to be chosen to be a part of it*. I *have learned a lot.”*

When asked about the appropriateness and feasibility of the model in focus groups midwives who facilitated the groups described how group postpartum and well-child care was perceived to be more efficient and effective than individual care. A midwife from Clinic B expressed their perceptions about the participants experience, saying:

Women are happy because postnatal groups are a one stop shop, they can get their vaccines, family planning, medications, be assessed, get health education all in one place instead of us telling them to go here, go there on this day and then there on that day, so they are very happy.

A midwife from Clinic B compared usual care to the group model. They explained:

What we are doing now is not working, we need to adopt this model of care because women are learning, when I asked what they had learned last session they were able to repeat back to me and I knew they had learned. When lecturing not sure who is listening or who is picking up on the information given.

Both midwives and HSA facilitators expressed appreciation for the model and that they felt that women would benefit from this model of care. They noted its potential to lead to better outcomes and that it should be adopted by the Malawian health care system. A midwife from Clinic B said, *“This is a good model because when women come for postnatal checks they only focus on the baby so we miss a lot of things. This model will lead to better outcomes.”* Several facilitators also explained that experiencing group healthcare also affected how they feel about their current work. An HSA from Clinic C said, *“I enjoyed the sessions because at the under 5 clinic I have no time to engage with women and* I *think that women will benefit from groups.”* Similarly, an HSA from Clinic B noted that, *“There was very active participation, so it made facilitation easy Lecturing does not help people learn, this does, and it should be adopted.”*

Women were asked to attend two sessions and 90% of women (n = 36) returned to participate in the second session. Those who were unable to return (n = 5) offered several explanations for not returning including death in the family (n = 1), conflicting schedules (n = 2), and miscommunications about the date and time of the second group meeting (n = 2).

### Fidelity

Fidelity to the group care model was high (Table 4) across all 18 sessions completed at the three participating clinics. Average clinic preparation scores ranged from 83 to 100 percent as did the community building scores. Community building was not observed at only one of the 18 group sessions completed.

Fidelity was good for facilitators and women conducting clinical and self-assessments within the group space, 83 percent of sessions attained the fidelity threshold (a score of 4 out of 4). Women responded positively to self-assessment including learning about blood pressure screening, weight for themselves and their infant, and height and head circumference for their infants. At one post-session focus group a mother stated, *“I was happy when doing the self-assessments.”* In most groups, the HSA or midwife facilitators would teach one woman how to do the self-assessments and then ask that woman to teach the next woman (e.g., women learned to do a blood pressure reading and did them for each other). When the women returned for the second session, the time taken for assessments was reduced and the midwife and HSA could focus on the clinical assessments. The fidelity checklist demonstrated variation in implementation of the health assessments by facilitators, for example some did full head to toe assessments while others did focused assessments (e.g. focusing on specific areas of the body or identified problem areas) of the woman and infant. Two of the three clinics used a privacy screen in the group space while conducting health assessments. Only two sessions took longer than the allotted 3045 minutes for health assessments, with the longest time spent on assessments being 49 minutes. On average health assessments including both self-assessments and clinical assessments of the woman and infant took 31 minutes, consistent with the facilitator’s implementation guidelines. In focus groups after sessions, facilitators described some challenges to health assessments and requested the following materials to run groups: stethoscopes (they are required to get their own stethoscopes and are not provided by the clinic), a separate assessment table for infants, a training video for focused health assessments for women and infants, a standardized way to document postpartum assessments, and new infant weight scales.

For interactive learning, average fidelity across sessions (scoring > 6 out of 8) ranged from 83 to 100 percent for the three clinics. Each group session is intended to start with an opening activity that includes a mindful breathing exercise. Research assistants noted a sense of relaxation after the group completed these opening activities, especially within the first sessions when women were not familiar with the integrated model. Some group sessions scored lower in this domain due to some women arriving late to group, so facilitators were unable to conduct all health assessments with women/infant dyads before starting the health promotion discussions and activities but were able to complete them at the end of the session. In focus groups after the sessions, women described facing transportation challenges and so thus arrived late to group sessions. In three of the group sessions, co-facilitators after reviewing the facilitator’s guide during their preparation for the session decided to let the women choose two of the three to four health promotion topic activities that interested them of those that were included in the session as they stated they felt they would not have enough time to cover all the topics. In five group sessions, co-facilitators did not have time to cover all the outlined session topics within the two-hour allotment.

Fidelity for environment and logistics average across sessions ranged from 50 to 100 percent (scores > 5 out of 6) for the clinics. For example, in Clinic A, one room was attached to a room that was used for other services, so people came in and out which was disruptive at times. In two group sessions due to competing clinical demands facilitators had to leave the session to attend other clinic duties. Two group sessions were disrupted because more women wanted to participate than were able to and were knocking on the door to try and enter.

The group dynamics average scores ranged from 13–18 (on a scale of 0–18) with a mean of 16.33, indicating that most women were highly engaged in the health assessments and interactive learning.

Co-facilitation skills average ranged from 4–12 (on a scale of 0–12) with a mean of 9.28. In one-third of group sessions, co-facilitators had either not prepared or had partly prepared for the group which led to disorganized discussion and lower scores in this fidelity domain. When asked why they hadn’t prepared during the facilitator’s focus groups after the sessions, most co-facilitators reported they did not receive sufficient time to prepare due to competing priorities or miscommunications as to the day they were going to be facilitating the groups. Other facilitators reported the need for additional practice with facilitation and the content. Co-facilitators’ use of strategies taught in the training such as asking open-ended questions and using the Centering-based facilitation strategy “acknowledge, refer, and return”(23, 30) varied among group session which also led to lower scores in this domain. However, in two-thirds of the groups, co-facilitators were well prepared, and facilitators used the strategy “acknowledge, refer, and return” during the discussion period of the group session. In just over half of the group sessions’ facilitators used open-ended questions in group discussion. These skills were all measured in the co-facilitation skills domain of the observation checklist.

The group care delivery domain captured the delivery of the content during the sessions. For example, whether the session was implemented as a didactic class/lecture or facilitated discussion, what was the level of group member engagement during the session, and what proportion of time did facilitators speak compared to women. These scores ranged from 7–12 (on a scale of 0–12) with a mean of 10.44. Only one of the 18 sessions was reported as more like a class/lecture than a discussion, the remaining sessions were described as half class/lecture and half discussion or mostly like a discussion. Sixteen sessions were rated by research assistants as having medium to high or very high levels of engagement. In 7 of the 18 groups, facilitators spoke more than women during the sessions, and in 11 group sessions facilitators and women either spoke equal amounts of time or women spoke more than facilitators. These were all measured in the group care delivery domain of the observation checklist.

The total fidelity observation checklist scores across the 18 sessions and three clinics were high (> 52 out of 65) with a mean of 55.89 and all meeting the threshold for acceptable fidelity. Clinic C had the highest implementation fidelity score.

When asked about their learning experience in the focus groups, women expressed how much they learned. A woman from Clinic A said, “*I felt that it’s different from what I normally know just one nurse in a hurry I felt in a circle I learned a lot. Some things I might not have learned otherwise.”* Another woman from the same group said, *“Normally I learn about one topic, but here we talked about many topics, mental health, danger signs, breastfeeding, I learned a lot in a small period of time.”*

Women discussed the topics covered at the two sessions and learning included how to do self-assessments (i.e., weighing themselves and their baby, how to take a blood pressure reading, and measuring their infant’s height and head circumference), how to breastfeed and exclusive breastfeeding. Other topics included the importance of blood pressure, vaccinations, and the diseases they protect against. A woman from Clinic B said, “*I am happy to learn the vaccine schedule.”* Several women reported learning about hypertension. A woman from Clinic C explained, “*I am happy to have learned about high blood pressure, now I know about hypertension and would like to continue with groups so that I can keep learning.”*

Other topics they described as being covered included nutrition for both mother and baby, when to introduce solid foods; danger signs of serious health conditions and when to go to the clinic; HIV and prevention of mother-to-child transmission; child health and development; and how to deal with stress. A woman from Clinic A linked extreme stress to when to seek care when she said, “*I learned about emotional health, marital problems cause stress, and that you should talk to other people, but if you see that your stress is extreme you should go to the hospital.”* They also reported learning what to do when a child is sick; hygiene and sanitation (e.g., latrines at home); and strategies to include men in their daily activities. A woman from Clinic C noted how the activity about asking for help affected her. She said,” *The male involvement activity was good because I take it as normal that I have to do everything, now I know I can ask for help*.” Additional health promotion topics included family planning; growth monitoring; mental and emotional health; relationship conflict management; and cervical and breast cancer. During two separate group sessions women described their cervical cancer screening experiences and encouraged the women who had not been screened to go for their screening.

### Health issues identified and services received within the integrated model

During each session the research team noted as a part of the fidelity observation checklist if midwives identified and/or treated health concerns during health assessments and if any services were received at the end of sessions e.g., infant vaccinations. In total 51 people (including both women and infants) over the course of the 18 group sessions had a health issue that was identified and treated during health assessments or received a health care service or were referred to other providers for care at the end of the group session. Health issues identified among women included: high blood pressure, perineal pain, difficulty breastfeeding, and finger pain. The most common health issue among women was high blood pressure. Health issues identified among infants included: skin rash, cough or flu-like symptoms, abscess, inadequate weight gain, inguinal and abdominal hernias, and a skin mass. The most common health issue among infants was cough and flu like symptoms.

Services that were received within the group space for women included: family planning, mosquito nets (for the prevention of malaria), medications (e.g., anti-hypertensives), and one was admitted for observation due to difficulties breastfeeding. The most common service received by women within the group space was family planning with the majority receiving Depo Provera. Services that were received within the group space for infants included: vaccinations, medications (e.g., antibiotics), and enrollment in a nutrition program for a malnourished child. Vaccines were most common service provided to the infants.

## Discussion

Eighteen integrated group postpartum and well-child care sessions were completed as part of this pilot. Both women and facilitators perceived the model to be highly acceptable, appropriate, and feasible to implement at clinics. Co-facilitators were also able to maintain fidelity to the three core components of the group care model, healthcare in a group space, interactive learning, and community building. Acceptability, appropriateness, and feasibility are considered “leading indicators” of implementation success (21). All facilitators found group care to be highly acceptable, appropriate, and feasible within their clinical setting, women had more variability in their evaluation of group sessions, this variability could be due to facilitator’s level of experience with content and skills with facilitating groups, transportation difficulties, or personal preferences for type of care they prefer for themselves and their infant. The implementation outcomes evaluated in this study serve as indicators of implementation success and set the stage for future research to test the effectiveness of integrated group postpartum and well-child care (21).

When adapting and implementing a Centering-based group care model in a new context, maintaining fidelity to its core components is essential to ensure that the features associated with the model’s effectiveness are retained (31). Model fidelity is important to monitor as it allows for better evaluation of the impacts of group care in the postpartum period (31). Fidelity scores varied across domains and clinics, the environment and logistics conducive for group care was the most challenging, and fidelity to community building and group dynamics was consistently high across clinics. Studies of group well-child care highlight the importance of community building and group dynamics in the postpartum period as it has been shown to foster partnerships between families, clinicians, and communities and is a key component in addressing health related social needs (11, 32–34). Clinic C demonstrated the highest model fidelity score as well as has the highest ratings of acceptability, appropriateness, and feasibility. Some factors that might have contributed to this are institutional leadership and a group care champion (11, 35), as one of the midwives facilitating was the in-charge of the clinic and was an experienced group ANC facilitator and trainer of facilitators.

Health issues identified for women that participated in group sessions highlight the unmet health needs occurring in the 12 months postpartum which emphasizes and justifies the need for high quality postpartum care. Non-communicable diseases (NCDs) are a significant contributor to maternal morbidity and mortality and the current state of maternal health care does not adequately identify or treat them as a part of routine care (36). Hypertension disorders in pregnancy are one of the leading causes of maternal morbidity and mortality (2, 5) and puts women at risk for chronic disease and long-term health consequences. Hypertension was the most common health issue identified during the women’s health assessments. Studies show a high prevalence of hypertension among women in the postpartum period in some African countries and missed opportunities to identify and treat it in the current state of postpartum care (37–39). Studies in Malawi specifically reveal high prevalence of and risk for hypertension and the need for innovative interventions to prevent, treat, and control hypertension (40). During the self-assessments within the group sessions, co-facilitators were able to identify and treat hypertension and schedule follow-up as needed, demonstrating that group care is able to increase the quality of care women receive.

In addition to hypertension, opportunities exist within group care to increase screening for other NCDs such as diabetes, anemia, and mental health. Prevalence of postpartum depression among women in Malawi is estimated to be 19.8% (41), highlighting the need for targeted screening and preventive measures to support women experiencing it. Group care provides an opportunity to fill a gap in the care continuum, identify and treat NCDs and other health issues, and assist in the transition from maternity care to primary health care services (36).

The WHO advocates for integrating health services as they have the potential to directly enhance wellbeing, improve access to services, improve health outcomes, and enhance health equity (42). Both women and facilitators described group care as an efficient way to deliver and receive health promotion education as well as health care services in one visit for both the woman and infant. The current state of postpartum and well-child care in Malawi requires women in the postpartum period to travel to the clinic multiple days to receive care and services for themselves and for their infants. This places a burden on women who often face long travel times, transportation difficulties, and missed work opportunities. The most common services received within the integrated group care session was family planning for women and vaccinations for infants. Thus, extending group care from into the postpartum period has the potential to further increase rates of family planning use.

During each group session all infants received their scheduled vaccines if they were due to receive them. Based on these results, we hypothesize that group care could maintain vaccine uptake, as infants were able to receive them at the end of the visit without extra waiting time. This is especially important since vaccines are important drivers in reducing infant morbidity and mortality. This is particularly important in Malawi, a country that recently experienced an outbreak of polio, a preventable childhood disease (43). Within the group session women also received assistance with difficulties with breastfeeding, nutritional education, and referrals to malnutrition programs where necessary employing both direct and indirect evidence-based and effective interventions to combat maternal and child malnutrition (44).

Integrating health services increases coordination across the care continuum to improve maternal and child health outcomes and there is some evidence that it is also cost effective (42). While some models of care throughout the African continent integrate aspects of maternal health care into pediatric care with success such as lactation support (45), and maternal mental health screenings (46), very few provide a full integration of postpartum and well-child care as presented in this study (47, 48).

### Implications for future research and practice

Future studies can assess the effectiveness of the model on clinical outcomes for both women and infants. Recommendations from facilitators for improving the model included: to add screening tools to each session such as a desire for family planning and postpartum depression; add a “cheat sheet” at the beginning of each session for co-facilitators to provide a quick guide for each session’s content; refine some activities to improve understanding; and reduce the number of activities outlined in each session so that the length of the session remains within the designed time of 2 hours. Adding screening tools and a standardized way to document assessments to the model will allow for assessment of the model’s impact on NCDs such as mental health and hypertension. Further studies are starting to show persistent hypertension up to one year postpartum across settings so this will be an important outcome to monitor (39, 49, 50). In addition, future iterations of model implementation can focus on strategies to enhance fidelity such as a focus on training co-facilitators to deliver the model (51). Malawi has established a rigorous training program that can be used to bolster co-facilitator’s skills for both group ANC, postpartum, and well-child care (23). We recommend future research refine the fidelity observation checklist to test its association with outcomes and create a standardized tool to use across diverse settings. Finally, this study showed that institutional leadership and program champions are key to implementing the group care model (11, 35). Staff buy-in has also been noted as important factor for implementation success and sustainability of the model (35). These will be important factors to consider when considering the model’s level of sustainability in future research.

### Limitations

This study has several limitations. This study was carried out in clinics that were already familiar with the group care model. So, the results may look different if the system-level care delivery changes were not understood by clinic staff. Also, some of the timing and implementation data may be influenced by the small number of participants (given the number of small groups i.e., 2–3 dyads in a session). It is possible that the timing of the groups and the implementation outcomes here may be different had the groups been at full capacity. We relied on self-report measures which may introduce response biases. Our findings inform the implementation of group postpartum and well-child care at peri-urban and rural clinics in Malawi but may not be generalizable to other clinic populations. Our focus was on the evaluation of implementation outcomes, and we cannot comment on the effectiveness of this integrated group postpartum and well-child care model compared to usual care. Last, because we did not measure effectiveness, we were unable to link fidelity to health outcomes.

## Conclusion

We found that clinics in Blantyre District, Malawi were able to implement an integrated model of group postpartum and well-child care. Both women and facilitators of the model found it to be highly acceptable, appropriate, and feasible and described positive experiences conducting and participating in group sessions. Further, the facilitators demonstrated fidelity to the model. These promising results set the stage for rigorous research to examine the effectiveness of this model on maternal and child health outcomes in Malawi and elsewhere.

## Figures and Tables

**Table 1:** Description of the fidelity observation checklist

Domain	Description	Example Item, Type of Response	Number of items (score range)	Threshold for Achieving Fidelity
Clinic Preparation	To assess if the clinic is ready to start group on the scheduled time.	Was the room set up before the schedule meeting time? Binary yes/no	3 (0–3)	3
Community Building	To assess if women are socializing with one another.	Are women socializing with one another? Binary yes/no	2 (0–2)	2
Health Assessments	To assess if health assessments are done within the group space and if all women participate in selfassessments.	Did health assessments take place within the group space? Binary yes/no	4 (0–4)	4
Interactive Learning	To assess if the interactive learning occurs with opening and closing activities, in a circle, and after health assessments are completed.	Was discussion conducted in a circle? Binary, yes/no	8 (0–8)	≥6
Environment and Logistics	To assess availability and adequacy of space and supplies.	Was the session disrupted for any reason? Binary yes/no	6 (0–6)	≥5
Group Dynamics	To assessment engagement of women during group.	About how many group members engaged in the following: Shared ideas, feelings, experiences, Likert scale	6 (0–18)	≥15
Co-Facilitator Skills	To assess co-facilitators’ use of facilitation strategies taught in group healthcare training.	Facilitators asked open-ended questions, Likert scale	6 (0–12)	≥10
Group Care Delivery	To assess the overall delivery of group care and the level of connectedness among group members.	Overall, to what extent was the group session more like a class/lecture or more like a discussion? Likert scale	3 (0–12)	≥10
Total Fidelity Score			**0–65**	**≥52**

**Table 2:** Women/Infant Dyad Characteristics

Characteristic	N = 41 N (%)
**Women Age**	
< 20	11 (26.83)
> 20	30 (73.17)
**Marital status**	
Single	10 (24.39)
Married	31 (68.29)
Divorced/Separated	11 (26.83)
**Number of live children**	
1	21 (51.22)
> 1	20 (48.78)
**Infant Age**	
Mean	2 months
Range	1 day – 10 months

**Table 3:** Summary of Acceptability of Intervention Measure (AIM), Intervention Appropriateness Measure (IAM), and Feasibility of Intervention Measure (FIM) Results

	Clinic A N = 37	Clinic B N = 39	Clinic C N = 38	Total N = 114
Measure	Mean (SD)	Mean (SD)	Mean (SD)	Mean (SD)
**AIM**				
Facilitator	4.90 (.20)	4.79 (.35)	4.94 (.22)	4.88 (.26)
Mother	4.41 (.50)	4.69 (.39)	4.87 (.23)	4.66 (.43)
**IAM**				
Facilitator	4.79 (.38)	4.77 (.31)	4.81 (.39)	4.79 (.35)
Mother	4.21 (.65)	4.66 (.41)	4.85 (.25)	4.58 (.53)
**FIM**				
Facilitator	4.92 (.22)	4.75 (.34)	4.88 (.25)	4.85 (.28)
Mother	4.24 (.60)	4.59 (.42)	4.83 (.26)	4.56 (.50)

1 = Strongly disagree, 2 = Disagree, 3 = Neitsher Agree nor Disagree, 4 = Agree, and 5 = Strongly Agree

**Table 4:** Summary of Results from Fidelity Observation Checklists Across Clinics

Fidelity Domain	Clinic A n = 6	Clinic B n = 6	Clinic C n = 6	Total N = 18
	**Percent reaching fidelity threshold**			
Preparation	83.33	83.33	100	88.89
Community building	83.33	100	100	94.44
Health Assessments	83.33	83.33	83.33	83.33
Interactive Learning	83.33	83.33	100	88.89
Environment and Logistics	66.67	50	100	72.22
	**Mean (SD)**			
Group Dynamics	17 (2)	16 (2.10)	16 (2.28)	16.33 (2.06)
Co-facilitation Skills	8.83 (3.54)	8.33 (3.50)	10.67 (1.51)	9.28 (3.00)
Group Care Delivery	10.33 (1.36)	9.67 (2.80)	11.33 (2.50)	10.44 (2.50)
Total	55.83 (6.77)	53.17 (8.45)	58.67 (6.59)	55.89 (7.25)

## Data Availability

The datasets generated and/or analyzed during the current study are not publicly available to maintain confidentiality of participants but are available from the corresponding author on reasonable request.

## Abbreviations

AIM: Acceptability of Intervention Measure
ANC: Antenatal care
COREQ: Consolidated Criteria for Reporting Qualitative Research
FIM: Feasibility of Intervention Measure
HCD: Human-centered design
HIV: Human immunodeficiency virus
HAS: Health surveillance assistant
IAM: Intervention Appropriateness Measure
IRB: Institutional Review Board
LMIC: Low- and middle-income country
NCD: Non-communicable disease
WHO: World Health Organization
